# Characterizing post-extubation negative pressure pulmonary edema in the operating room—a retrospective matched case-control study

**DOI:** 10.1186/s13741-018-0107-6

**Published:** 2018-12-06

**Authors:** Pei-Hsin Tsai, Jen-Hung Wang, Shian-Che Huang, Yen-Kuang Lin, Chen-Fuh Lam

**Affiliations:** 1 Department of Anesthesiology, E-Da Hospital/E-Da Cancer Hospital, I-Shou University, No 1, Yida Road. Yanchao Dist., Kaohsiung city, 824 Taiwan; 2 Department of Anesthesiology, Buddhist Tzu Chi General Hospital, Hualien, Taiwan; 3 Department of Medical Research, Tzu Chi General Hospital, Hualien, Taiwan; 4 Biostatistics Center, Taipei Medical University, Taipei, Taiwan

**Keywords:** Upper airway obstruction, Respiratory complication, Extubation failure

## Abstract

**Background:**

Post-extubation negative pressure pulmonary edema (NPPE) is an uncommon but important anesthesia-related emergency presenting with acute respiratory distress and hypoxemia after removal of airway devices. This study investigated the incidence and associated risk factors for post-extubation NPPE during emergence.

**Methods:**

This retrospective, matched case-control study was conducted by reviewing the post-anesthesia records in Tzu Chi General Hospital, Taiwan. Patients reported of having acute hypoxemia (SpO_2_ < 92%) shortly after the removal of the endotracheal tube or supraglottic airway, associating with radiographic evidence of pulmonary edema and/or pink frothy sputum, were identified as definite NPPE cases. The potential risk factors were compared with the matched controls, who were randomly selected from the same database.

**Results:**

A total of 85,561 patients received general anesthesia with airway instrumentation during the 8.5-year study period. A total of 16 patients were identified as definite cases of NPPE. Compared with the matched controls (*n* = 131), males, active smokers, emergency operation, endotracheal intubation, use of desflurane, and prolonged operation time carried significantly higher risks of developing NPPE (*P* < 0.05). Multivariate logistic regression analysis illustrated that active smoking (AOR 7.66, 95% CI 1.67–35.3; *P* = 0.009) and endotracheal intubation (AOR 10.87, 95% CI 1.23–100; *P* = 0.03) were the two most significant independent variables of post-extubation NPPE.

**Conclusion:**

We present the first clinical comparative study demonstrating that the overall incidence of NPPE immediately after extubation in the operating room is 0.019%. Our results highlight that active smokers and patients receiving endotracheal intubation general anesthesia are associated with significantly higher risks of developing NPPE following extubation in the operating room.

**Electronic supplementary material:**

The online version of this article (10.1186/s13741-018-0107-6) contains supplementary material, which is available to authorized users.

## Background

Post-extubation negative pressure pulmonary edema (NPPE) or post-obstructive pulmonary edema (POPE) occurs following a large negative intrathoracic pressure generated by forceful inspiration against an obstructed airway, such as laryngospasm or mechanical obstruction (Lemyze and Mallat 2014). The generation of extremely high negative intrapleural pressure significantly increases the pulmonary capillary permeability and enhances venous return into the right heart, leading to fluid translocation from intravascular system to the pulmonary interstitium (Lang et al. 1990). During the emergence phase of anesthesia, NPPE most commonly happens in patients with acute laryngospasm following the removal of an endotracheal tube or supraglottic airways (Lorch and Sahn 1968; Ghofaily et al. 2013). The onset of pulmonary edema is usually rapid (within a few minutes after signs of upper airway obstruction) and presents with hypoxemia and acute pulmonary edema (radiographic changes in the chest and pink frothy hemoptysis) (Lemyze and Mallat 2014; Ghofaily et al. 2013). In general, NPPE is usually a benign condition typically resulting in full recovery within 12–48 h (Krodel et al. 2010). However, it can be a true post-anesthesia emergency that requires immediate tracheal re-intubation, and up to 50% of these patients are subjected to prolonged mechanical ventilatory support due to acute respiratory failure (Krodel et al. 2010; McConkey 2000).

The estimated incidence of post-extubation NPPE is approximately 0.01–0.1% during general anesthesia (Krodel et al. 2010; McConkey 2000; Deepika et al. 1997; Bhaskar and Fraser 2011). In a retrospective case-control study, NPPE were more frequently reported in healthy (ASA physical status I and II), middle-aged, and male patients (Deepika et al. 1997). Since NPPE is a rare post-anesthesia event, there are currently no large-scale comparative clinical studies available in the literature. In addition, the incidence and the precipitating factors of post-anesthesia NPPE are difficult to compute from the case series data or descriptive studies (Bhattacharya et al. 2016). Therefore, we performed a retrospective, matched case-control study to analyze the overall incidence and the associated risk factors of post-extubation NPPE during the emergence period of anesthesia from the database of post-anesthesia records in our hospital.

## Methods

### Patient database

**Fig. 1:**
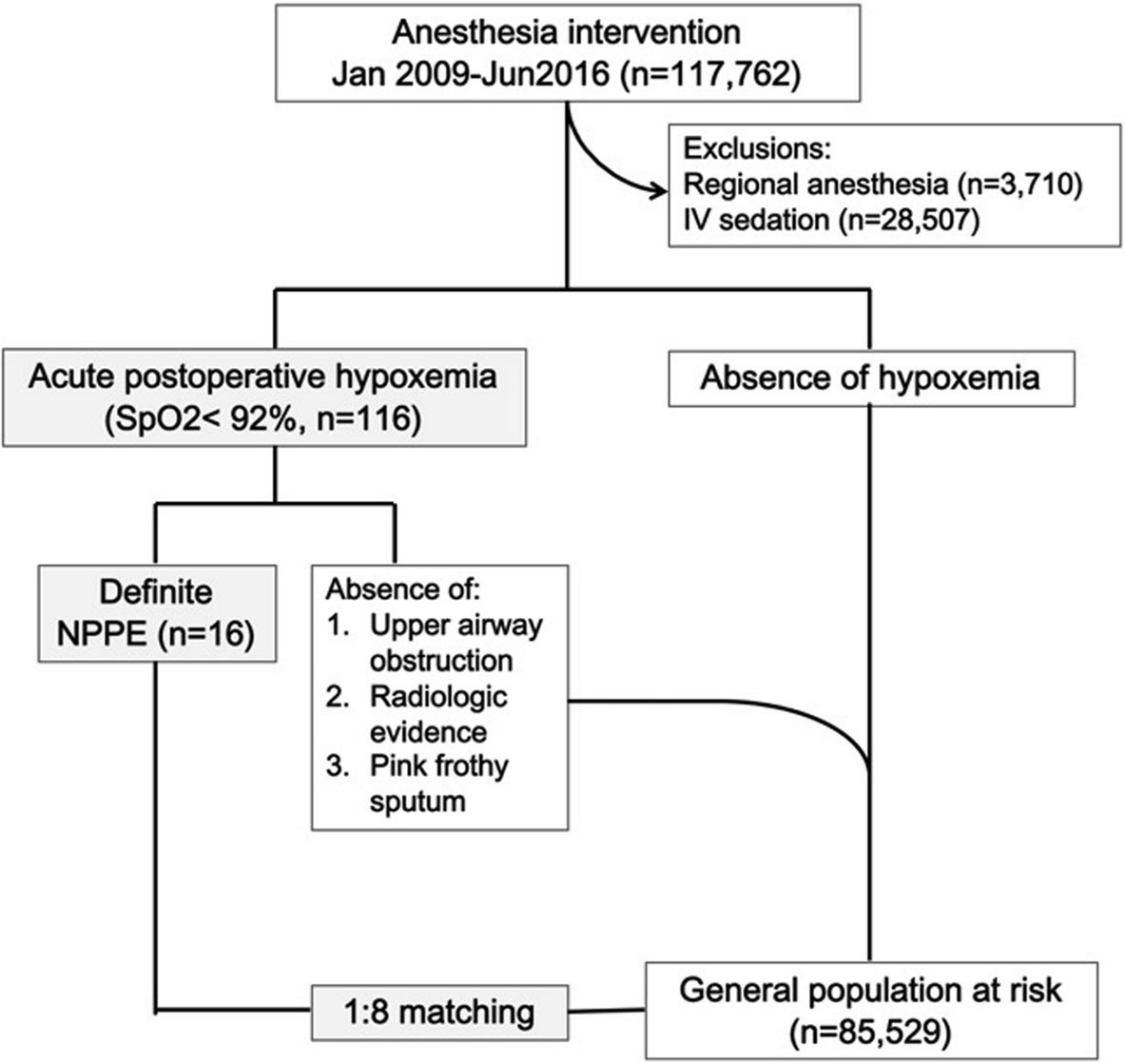
Study design and matching of case-controls

This retrospective chart review study carried out in a tertiary teaching medical center located at Hualien City of Taiwan that consists of 945 beds. The study was approved by the ethics committee and the institutional review board (IRB, Approval number IRB106-22-B), and the requirement for written informed consent was waived by the ethics committee. All surgical patients received endotracheal general anesthesia (ETGA) or laryngeal mask anesthesia (LMA) during 1 January 2008 to 31 May 2016 were included in this study, except for those who received intravenous sedation without airway instrumentation and removal of airway devices in medical care units other than operation room (Fig. 1). Active smoker was defined as a patient who actively smoked at least one cigarette a day within the preceding week of surgery (Warner et al. 2004).

### Definition of post-extubation NPPE

Post-extubation NPPE was defined as the development of acute hypoxemia (pulse oximetry, SpO_2_ < 92%) with witnessed signs of upper airway obstruction by the anesthetists after removal of an endotracheal tube or laryngeal mask airway in the operation room. Definite cases of NPPE should also coexist with evidence of chest radiography of newly occurred pulmonary edema and/or cough with pink frothy sputum (Ghofaily et al. 2013).

### Matched controls

Matched controls were surgical patients who received ETGA or LMA intervention without developing desaturation and clinical signs of pulmonary edema during the study period. These patients were randomly selected from the same database after matching with the calendar year of operation in a 1:8 ratio.

### Statistics

The potential categorical and numerical risk factors of occurrence of NPPE were compared with the matched case-control patients who did not develop NPPE by the chi-square and Wilcoxon signed-rank tests, respectively. A conditional logistic regression model was adopted to evaluate the association between these risk factors and NPPE. Statistical significance was accepted at a level of *P* < 0.05. All statistical analyses were performed using SAS 9.4 (SAS Institute, Cary, NC).

## Results

There were a total of 117,762 patients who received anesthesia management during the 8.5-year period in our hospital, and 85,545 of these surgical patients were anesthetized with an endotracheal tube (ETGA) or laryngeal mask (LMA) (Fig. 1). Since perioperative desaturation (SpO_2_ < 92%) is an imperative quality assurance parameter that has been consistently monitored in our hospital, all cases who developed desaturation during the emergence period were reviewed. A total of 116 patients were recorded to experience desaturation during the study period, and 16 patients were identified as definite cases of NPPE shortly after the removal of airway devices in the operating room (Table 1). Most of these patients (9/16) were transferred to the intensive care unit (ICU) after the unexpected event, and 1 patient eventually expired 50 days later due to uncontrollable postoperative pulmonary complication and development of multiple system organ failure (Table 1).

**Table 1:** Characteristics and outcomes of post-extubation negative pressure pulmonary edema

Patient ID	Gender	Age	BMI	ASA PS	Operation	Type of anesthesia	Anesthetics	Operation time (min)	Unit of transfer
1	F	57	21.9	II	Radiofrequency ablation of hepatoma	ETGA	Sevoflurane	200	ICU
2	M	22	17.9	I	Local flap	ETGA	Desflurane	115	PACU
3	M	64	31.6	II	Right parotidectomy + muscular rotation flap	ETGA	Sevoflurane	195	PACU
4	F	41	20.9	III	Gamma nail for fracture	ETGA	Sevoflurane	135	PACU
5	M	49	27.3	II	Left submandibular sialithotomy	ETGA	Desflurane	60	PACU
6	M	67	17.3	III	Bilateral mandibular condylectomy	ETGA	Sevoflurane	510	ICU
7	F	66	36.5	II	Laparoscopic appendectomy	ETGA	Sevoflurane	75	PACU
8	M	57	17.2	II	Right below knee amputation	LMA	Sevoflurane	195	ICU
9	M	19	31.1	I	ORIF + short leg splint	LMA	Sevoflurane	180	ICU
10	M	14	18.4	I	Laparoscopic appendectomy	ETGA	Desflurane	105	ICU
11	F	49	21.5	IIE	Repair of perforated gastric ulcer	ETGA	Sevoflurane	200	PACU
12	F	38	41.3	IIE	Cesarean section + tubal ligation	ETGA	Sevoflurane	135	ICU
13	F	62	23.1	II	Release of scar + flap	ETGA	Desflurane	140	PACU
14	M	44	30.7	II	Uvulopalatopharyngoplasty	ETGA	Desflurane	145	ICU
15	M	18	16.7	IIE	Laparoscopic appendectomy	ETGA	Sevoflurane	75	ICU
16	M	55	20.0	IIE	Segmental resection of small bowel	ETGA	Sevoflurane	210	ICU*

*ASA PS* American Society of Anesthesiologists Physical Status (“E” indicates emergency operation), *BMI* body mass index, *ETGA* endotracheal tube general anesthesia, *ICU* intensive care unit, *LMA* laryngeal mass anesthesia, *ORIF* open reduction and internal fixation, *PACU* post-anesthesia care unit. *This patient was admitted to the ICU after the development of negative pressure pulmonary edema and eventually expired on day 50 after ICU admission

Compared with the matched controls (*n* = 131), patients who developed post-extubation NPPE were younger (49.4 ± 19.7 vs 45.1 ± 18.1 years, respectively; *P* = 0.065), male gender predominant (61/131 vs 10/16, respectively; *P* < 0.001), and consisted of higher proportion of active smoker (26/131 vs 9/16, respectively; *P* < 0.001) (Tables 2 and 3). The body mass index (BMI) of the post-extubation NPPE patients was significantly lower, but levels of ASA physical status were not different between the two groups (Tables 2 and 3).

**Table 2:** Characteristic analysis of the categorical risk factors associated with post-extubation negative pressure pulmonary edema (NPPE)

Categorical variables	Case (*n* = 16)		Matched controls (*n* = 131)		*P* value
	*n*	%	*n*	%	
Male	10	62.5	61	46.6	< 0.001*
ASA PS					0.174
I–II	14	87.5	101	77.1	
III–IV	2	12.5	30	22.9	
Emergency operation					
Yes	4	25.0	16	12.2	0.003*
Anesthetic technique					< 0.001*
ETGA	14	87.5	85	64.9	
LMA	2	12.5	46	35.1	
Difficult airway					
Yes	0	0	17	13.0	< 0.001*
Type of inhaled anesthetic					0.016*
Desflurane	5	31.3	18	13.7	
Sevoflurane	11	68.7	107	81.7	
Active smoker					
Yes	9	56.3	26	20.0	< 0.001*

*ASA PS* American Society of Anesthesiologists Physical Status, *ETGA* endotracheal tube general anesthesia, *LMA* laryngeal mask anesthesia. Data were analyzed by chi-square test and are shown as number (percent). *P < 0.05 is considered statistically significant

**Table 3:** Characteristic analysis of the numerical risk factors associated with post-extubation negative pressure pulmonary edema (NPPE)

Numerical variables	Case	Matched controls	*P* value
Age	45.1 ± 18.1	49.4 ± 19.7	0.065
Body mass index	24.6 ± 7.6	25.1 ± 5.3	< 0.001*
Duration of operation (min)	167.2 ± 103.8	148.4 ± 92.1	0.022*
Intraoperative morphine (mg)	2.0 ± 2.9	3.1 ± 9.6	0.247
Intraoperative fluid (ml)	1456.3 ± 906.3	1040.4 ± 947.5	< 0.001*
Intraoperative urine output (ml)	256.3 ± 394.5	147.8 ± 286.8	0.004*
Estimated blood loss (ml)	156.3 ± 272.4	128.3 ± 272.8	0.091

Data were analyzed by Wilcoxon signed-rank test and are shown as mean ± SD. *P < 0.05 is considered statistically significant

The significantly higher proportion of patients who developed post-extubation NPPE received emergency operation (16/131 vs 4/19; *P* = 0.003), and the operation time was prolonged in these patients (148.4 ± 92.1 min vs 167.2 ± 103.8 min; *P* = 0.022) (Tables 2 and 3). With longer operation time, the total intravenous fluid administration, urine output, and estimated blood loss during the perioperative period were also increased in the post-extubation NPPE group (Table 2). The total doses of morphine administrated intravenously for perioperative pain control were not different (Table 2). In comparison to the matched case-controls, the statistical analysis showed that proportion of patients anesthetized with ETGA and use of desflurane were significantly increased in the post-extubation NPPE group (*P* < 0.001 and *P* = 0.016, respectively; Table 3). There were no significant differences between body sites of operation (such as upper airway surgery vs surgery on other body parts) and the post-extubation NPPE (Additional file 1: Table S1).

These patient’s characteristic and surgery-related and anesthesia-related risk factors that associated with post-extubation NPPE were further analyzed by multivariate logistic regression. Male gender and use of desflurane became not significantly different in the development of NPPE following the removal of the endotracheal tube and laryngeal mask (Table 4). Most importantly, active smoking [adjusted odds ratio (AOR) 7.66, 95% CI 1.67–35.3; *P* = 0.009] and endotracheal intubation (AOR 10.87, 95% CI 1.23–100; *P* = 0.03) were identified as the two independent variables that are significantly associated with the occurrence of post-extubation NPPE in the operating room (Table 4).

**Table 4:** Multivariate conditional logistic regression analysis

Risk factors	AOR	95% CI	*χ* ^2^	*P*
Gender				
Male vs female	1.31	0.24–7.12	0.09	0.75
Age (years)	1.02	0.98–1.05	0.96	0.33
Smoking				
Yes vs no	7.68	1.67–35.36	6.85	0.009*
Type of anesthetics				
Des vs Sevo	0.73	0.17–3.14	0.18	0.67
Anesthesia technique				
ETGA vs LMA	10.87	1.23–100	4.60	0.03*
Duration of operation (min)	1.00	0.99–1.02	0.11	0.73
Intraoperative fluid (ml)	0.99	0.99–1.00	1.00	0.32

Multivariate conditional logistic regression model was adopted to evaluate the association between the potential risk factors and the development of POPE. *AOR* adjusted odds ratio, *CI* confidence interval, *Des* desflurane, *ETGA* endotracheal general anesthesia, *LMA* laryngeal mask anesthesia, *Sevo* sevoflurane, *χ*^2^ chi-square analysis. Smoking was defined as actively smoking at least one cigarette a day within the preceding week of surgery. *P < 0.05 is considered as statistical significant

## Discussion

The foremost important clinical message addressed in this study is that post-extubation NPPE is a relatively rare complication in the operating room with an overall incidence of 0.019% (≈ 19 cases in 100,000 general anesthesia with airway instrumentation), but it results in increased extraneous medical cost, as 56% of patients who developed unexpected NPPE after extubation were admitted to ICU for postoperative care, and it also engendered to a case of mortality in this study. Therefore, the recognition of the associated risk factors is essential to enhance our ability to prevent the development of NPPE following extubation of airway instruments in the operating room during the emergence period.

Dr. Deepika et al. reported one of the largest scale case series studies in patients developed NPPE in the operating rooms, post-anesthesia care units (PACU), and ICU after surgery (Deepika et al. 1997). The overall incidence of post-extubation NPPE was 0.094% (30 cases in 31,826 surgical patients) in their clinical case review (Deepika et al. 1997). A similar incidence of 0.084% (14 cases in 16,653 surgical patients) was reported during orthopedic surgery that eventually developed post-extubation NPPE (Patton and Baker Jr 2000). In light of the total number of cases reviewed, site of extubation, defined inclusion criteria, and study population, we believe that the overall incidence of 0.019% reported in our study is reasonably close to these previous case review studies. Hence, the results of our study are applicable to the general surgical population who received anesthesia with ETGA or LMA and anticipated to extubate in the operating room after surgery.

Several perioperative risk factors of post-extubation NPPE have been proposed, including patients of younger age, male, generally healthy (ASA I–II), difficult intubation, use of irritant volatile agents, operation on the head and neck region, obese, and recent upper airway infection (Lang et al. 1990; Lorch and Sahn 1968; Ghofaily et al. 2013; Krodel et al. 2010; McConkey 2000; Deepika et al. 1997; Bhaskar and Fraser 2011; Scarbrough et al. 1997). However, none of these factors has been characterized in a comparative analysis manner. In this study, we randomly selected non-case controls from the same database after matching with the calendar year of operation, as different time points of operation over the 8.5 years of study period might confound the outcome analysis (Rose and Laan 2009). Compared with the matched controls, univariate analysis demonstrated that the numbers of male gender, active smoker, and lower BMI (all *P* < 0.001) were significantly higher in the post-extubation NPPE group, while the incidence was marginally increased in younger age (*P* = 0.065), and there was no difference in ASA physical statuses (*P* = 0.174). These findings are consistent with the general idea that young, healthy males are more likely to generate greater inspiratory force to induce extraordinarily high negative intrapleural pressure for the shifting of lung interstitial fluid when the upper airway is occluded secondary to laryngospasm or obstruction following the removal of airway device (Krodel et al. 2010; Scarbrough et al. 1997). In line with some previous studies, our analysis did not find obesity contributes to increased risk of NPPE (Mulkey et al. 2008) but speculated that smoking is strongly associated with the occurrence of post-extubation NPPE (Mulkey et al. 2008). Smoking increases the upper airway reflex sensitivity (Erskine et al. 1994) and has been widely recognized as an independent risk factor of postoperative adverse events on the upper airway, which carries relative risks of 1.8 in all smokers and 2.3 in young smokers (16–39 years) (Schwilk et al. 1997).

The associations between post-extubation NPPE and anesthesia-related variables were analyzed in this study. Anesthesia with endotracheal intubation (ETGA) and the use of desflurane as an inhaled anesthetic agent are highly correlated with the occurrence of NPPE after extubation. The study did not find patients who eventually developed post-extubation NPPE and were documented as cases of difficult airway during the induction phase of anesthesia. Anesthesia with a laryngeal mask has been proven to reduce the incidence of laryngospasm during the emergence phase of anesthesia in the general population (Yu and Beirne 2010) and infants (Drake-Brockman et al. 2017). Therefore, more invasive airway manipulation using an endotracheal tube is inevitably associated with a higher incidence of laryngospasm and, subsequently, the development of post-extubation NPPE compared with LMA. Inhaled desflurane is generally considered to induce more upper airway response than sevoflurane during the perioperative period (Arain et al. 2005). However, recent studies indicate that the respiratory events were not increased with the use of desflurane considering the concentrations of anesthetic gas are carefully titrated (Stevanovic et al. 2015). In fact, cigarette smoking, but not the choice of anesthetic agents (desflurane vs sevoflurane), increases the risk of respiratory complications after operation (McKay et al. 2006). Furthermore, desflurane may provide a more rapid emergence and recovery profile that enhance its application in prolonged operation with anticipating extubation in the operating room (McKay et al. 2006). Therefore, the direct relationship between a particular anesthetic gas and post-extubation NPPE is not possible to establish using the univariate analysis. Our study also investigated the potential contribution of surgical-related factors and identified that emergency operation and prolonged operation time are the two notable risk factors associated with the development of post-extubation NPPE. Interestingly, there were four emergency operation patients who developed NPPE after extubation, and they all received ETGA anesthesia, reinforcing our above findings that LMA anesthesia is less irritable in the airway. The site of operation is another risk factor that has been frequently discussed, as some studies found NPPE occurred more often in the head and neck surgery (Deepika et al. 1997), while others did not (Mulkey et al. 2008). In the present study, we did not find significant effects of surgical sites on the occurrence of NPPE.

Models of multivariate logistic regression were used in this study to identify the dominant variables associated with post-extubation NPPE. Only two measurement variables were found as strong independent risk factors of NPPE in the operating room, namely active smoking and ETGA, with AORs of 7.66 (95% CI 1.67–35.3; *P* = 0.009) and 10.87 (95% CI 1.23–100; *P* = 0.03), respectively. Although the other variables failed to demonstrate statistically significant differences in multivariate regression analysis, we specify that male gender, younger age, and prolonged operation should be considered as precipitating factors in the development of NPPE in the operating room, especially in active smokers and patients receiving endotracheal intubation.

There are several limitations in this study. First, the retrospective study design limits the ability to establish any direct causal relationships between the measured variables and post-extubation NPPE. Since the incidence of post-extubation NPPE is relatively very rare, matched case-control study design rather than propensity matching was used to identify the associated risk factors, resulting in a large range of data variance. Nevertheless, propensity matching of patient or surgical characteristics (such as age, gender, ASA classification, or types of surgery) might in fact introduce confounding effects to the analysis (Pearce 2016), as these characteristics could be the independent risk factors of NPPE. This study is also subject to potential missing cases from medical chart review. However, perioperative hypoxemia (SpO_2_ < 92%) is one of the key clinical parameters for monitoring of anesthesia quality assurance in our institute with an overall incidence of 0.13% in patients who received ETGA or LMA anesthesia. Therefore, it would be very unlikely that clinically severe hypoxemia cases after extubation in the operating room were undetected in our anesthesia records. Nevertheless, those who developed subclinical degrees of NPPE (SpO_2_ ≥ 92%) were not included in this report, as chest radiography is not routinely taken in the operating room or PACU with the absence of signs of clinical hypoxemia. Although smoking has been identified as an independent risk factor for post-extubation NPPE, duration of smoking and the amount of cigarettes consumed each day were not taken into the analysis. Finally, this study did not include other potential risk factors, such as recent symptoms of upper airway infection or asthma, as these information were not comprehensively recorded in each pre-anesthesia assessment file.

## Conclusion

We present the first comparative clinical study reporting the incidence and risk factors for NPPE during the emergence phase of anesthesia in the operating room. The overall incidence of post-extubation NPPE in the operating room is about 0.019%. Multivariate logistic regression analysis indicates that active smokers and anesthesia with ETGA are the two most important independent risk factors for developing NPPE in the post-anesthesia setting. We also highlight that male gender, younger age, and prolonged operation time should be considered as precipitating factors in the development of NPPE in the operating room, especially in active smokers and patients receiving endotracheal intubation.

## Additional file

Additional file 1: **Table S1.** Characteristic analysis of the body parts (sites) of operation associated with post-extubation negative pressure pulmonary edema (NPPE). (DOCX 65 kb)

## Abbreviations

AOR: Adjusted odds ratio
ASA: American Society of Anesthesiologists
BMI: Body mass index
ETGA: Endotracheal intubation general anesthesia
ICU: Intensive care unit
IRB: Institutional review board
LMA: Laryngeal mask anesthesia
NPPE: Negative pressure pulmonary edema
POPE: Post-obstructive pulmonary edema
