# Supplementary material for: Characterizing post-extubation negative pressure pulmonary edema in the operating room—a retrospective matched case-control study
Source: Perioper Med (Lond). 2018 Dec 6;7:28. doi: 10.1186/s13741-018-0107-6 (PMC6282297; doi:10.1186/s13741-018-0107-6)
Supplement: Supplementary file 1 — Table S1. Characteristic analysis of the body parts (sites) of operation associated with post-extubation negative pressure pulmonary edema (NPPE). (DOCX 65 kb) [file 13741_2018_107_MOESM1_ESM.docx]

**Table S1 Characteristic analysis of the body parts (sites) of operation associated with post-extubation negative pressure pulmonary edema (NPPE)**

| **Site of surgery** | **Case** | | **Matched controls** | | **P value** |
| --- | --- | --- | --- | --- | --- |
|  | n | % | n | % |  |
| Laparoscopy, abdominal | 1 | 6.25 | 4 | 3.05 |  |
| Laparotomy, abdominal | 2 | 12.5 | 8 | 6.11 |  |
| Breast surgery | 0 | 0 | 15 | 11.45 |  |
| Spine surgery, cervical | 0 | 0 | 1 | 0.76 |  |
| Spine surgery, lumbar | 0 | 0 | 6 | 4.58 |  |
| Extremities, lower | 3 | 18.75 | 30 | 22.90 |  |
| Extremities, upper | 0 | 0 | 5 | 3.82 |  |
| Head-and-neck | 3 | 18.75 | 15 | 11.45 |  |
| Lower abdomen | 5 | 31.25 | 20 | 15.27 |  |
| Upper airway | 2 | 12.5 | 27 | 20.61 |  |
| **Total** | 16 | 100 | 131 | 100 | 0.692 |

Data were analyzed by Chi-square test and are shown as number (percent). *P< 0.05 is considered statistically significant.
